# Effects of religious orientation and state secularism on pre-implantation genetic diagnosis

**DOI:** 10.1016/j.heliyon.2020.e04798

**Published:** 2020-08-29

**Authors:** Zira Hichy, Federica Sciacca, Graziella Di Marco, Concetta De Pasquale

**Affiliations:** Department of Educational Sciences, University of Catania, Italy

**Keywords:** Psychology, State secularism, Religious orientation, Pre-implantation genetic diagnosis

## Abstract

This study aimed to test the associations of religious orientation (extrinsic, intrinsic, and quest) and secularism of state with individuals' attitudes towards the pre-implantation genetic diagnosis of embryos. Moreover, we tested the mediating effects of secularism of state on the relationship between religious orientations and attitudes towards this issue related to embryos. Participants were 312 Catholic Italians that completed a questionnaire containing measures of investigated constructs. Results showed that attitude towards pre-implantation genetic diagnosis negatively correlated with intrinsic religious orientation and positively with extrinsic religious orientation and secularism. Moreover, results indicated that secularism mediated the relationship between extrinsic and intrinsic orientation and attitude towards pre-implantation genetic diagnosis. Taking together these results indicate that real endorsement with religion is associated with the refusal of pre-implantation genetic diagnosis because intrinsic religious orientation is related to the desire for state laws to follow religious principles; on the other hand, the use of religion for utilitarian reasons is associated to the acceptance of pre-implantation genetic diagnosis in order to have a religious state and then maintain Catholics’ privileges.

## 1 Introduction

In various countries, there is an ongoing debate about technologies related to embryos, including pre-implantation genetic diagnosis. The pre-implantation genetic diagnosis (PGD) is the genetic profiling of embryos before their implantation in the uterus, used for the screening of specific genetic diseases. This technique raised many ethical discussions in various countries (Baldwing, 2009; Robertson, 2003) and is not allowed everywhere. Studies showed a wide approval of PGD for medical purposes, such as hereditary cancer, and a general denial for other purposes, such as sex-selection (Bucchi et al., 2006; Meister et al., 2005; Rich et al., 2014). Most of the studies investigating attitudes toward PGD were carried out with participants who may need PGD and showed that the principal predictor for PGD acceptance is the subjective familiarity with genetic diseases (Wah Hui et al., 2002; Hershberger and Pierce, 2010). Despite it could be important to investigate attitudes towards PGD in the general population, few studies were conducted. It is important to analyze the attitude of the general population regarding PGD because it can influence its regulation. In this sense, an emblematic case was the referendum held in Italy in 2005, aimed at abrogating a law forbidding PGD, that was invalidated because people didn't go to vote and the voter turnout was below the 50% threshold. Therefore, as we can see, the absence of support in the general population might have strong effects on the regulation of PGD. The few studies carried out in the general population showed that favor towards embryo-related technologies are negatively related to religious beliefs (Hichy and Di Marco, 2014; Finck et al., 2006; Nisbet, 2005; Pardo and Calvo, 2008) and positively related to support for a secular state (Di Marco, Hichy, Coen and Rodriguez-Espertal, 2018; Hichy and Di Marco, 2014).

With regards of religion, most of the studies investigating this variable measured religiosity as religious practices or as a general involvement in religion, but these measures do not take into account the different ways in which people can be religious; moreover, to our knowledge, no study concerning religious oyze the effecrientations and attitude towards PGD has previously been carried out in Italy. For this reason, the principal aim of this study was to analts of different ways of being religious, that is the effects of religious orientation (extrinsic, intrinsic, and quest; Allport and Ross, 1967; Batson et al., 1993), on attitude towards PGD.

Whenever discussions concerning the regulation of issues that might involve a violation of religious principles the debate on the secularism of State arises. Indeed, studies showed that the rejection of a state free from religious influence involves the refusal of PGD, embryonic stem cell research, same-sex marriage, and adoption by same-sex couples (Di Marco et al., 2018; Hichy and Di Marco, 2014; Hichy et al., 2015a, Hichy et al., 2015b). For these reasons is important to analyze the effects of people's attitude towards a secular state, indeed, the second aim of the study was to test the effect of secularism on the relationship between religious orientation and attitude towards pre-implantation genetic diagnosis.

### 1.1 Religious orientations

As we have seen, studies showed that religion negatively influences attitude towards PGD, however, to our knowledge, no study analyzed the effects of different ways of being religious on attitude towards PGD.

Religiosity takes on a significant part in determining people's individual and social life (Roccas and Elster, 2014), and it is difficult to provide a univocal definition that includes all forms that religiosity can take (Batson and Ventis, 1982). Indeed, literature distinguishes three diverse ways of being religious that correspond to three diverse orientations: extrinsic, intrinsic, and quest (Allport, 1950; Allport and Ross, 1967; Batson et al., 1993). Intrinsic orientation states to a mature and internalized form of religiosity; people embracing this orientation consider religion as the principal reason in their life and completely follow religious principles (Allport and Ross, 1967). Intrinsic orientated people, in line with religious teaching, seem to be tolerant and compassionate, but this apparent tolerance does not always translate into positive attitudes; indeed, some studies have shown that intrinsic orientation is connected to low levels of open-mindedness and high levels prejudice (Batson and Ventis, 1982; Bosetti et al., 2011; Duck and Hunsberger, 1999; Hunsberger and Jackson, 2005).

Extrinsic religiosity is an immature form of religion, characterized by utilitarian and instrumental values, and based on impulsiveness and self-gratification (Allport and Ross, 1967). Extrinsically oriented individuals use religion to achieve non-religious ends (e.g., to obtain protection, relief, and to create good social relationships); the religion is held just because it serves other, more ultimate interests and shaped to adapt to other needs. Research has shown that extrinsic religious orientation negatively affects open-mindedness and positively affects prejudices (Batson et al., 1993; Bosetti et al., 2011; Hunsberger and Jackson, 2005).

Intending to identify a religious orientation that was linked to universal compassion and tolerance, Batson e colleagues (Batson et al., 2001; Batson et al., 1993) proposed a third type of religiosity, called quest orientation. This orientation refers to a specific dimension of religiosity, assumed as an open and interrogative approach to religion; people endorsing quest religious orientation recognize that they “do not know”, and will perhaps never know the truth on religious issues. Quest orientation correlates positively with cognitive complexity, doubt, open-mindedness, and universal compassion, and negatively with prejudice (Batson et al., 2008; Batson et al., 2001; Batson et al., 1986; Batson et al., 1978).

Regarding the relationships between religious orientations and attitude towards PGD, we hypothesized that this attitude should be negatively affected by intrinsic religious orientation, because this procedure violates religious principles. On the other hand, attitude towards PGD should be positively affected by quest religious orientation, because this orientation is related to openness and changing viewpoints (Hunsberger and Jackson, 2005); it is, therefore, possible that quest orientation should make people more able to understand the desire to have healthy children, although this may cause the destruction of embryos. Concerning extrinsic religious orientation, we hypothesized that it would not correlate with attitude toward PGD, because this orientation, instead of representing a genuine faith, denotes the use of religion just to achieve other ends. Then, unless PGD interferes with the achievement of other goals, there should be no relationship between extrinsic orientation and PGD.

### 1.2 Secularism of state

Concerning secularism, literature provides various views of secularism. For example, some scholar defines two forms of secularism: historical (based on equality and freedom of conscience) and new secularism (based on separation and neutrality; see, Roebroeck and Guimond, 2016). In opposition, other scholars define a unique form of secularism centered on the absence of discrimination based on religious convictions (Pena-Ruiz, 2003). Other scholars define the secularism based on its strength, identifying hard (explicitly atheistic states) vs soft secularism (states that have a purely formal relationship with the church; Kosmin, 2007). Despite these different views, secularism might be generally defined as the separation between the religious and the governmental dimension, that is the absence of religious involvement in government matters and the absence of political involvement in religious matters. Secular states have little or no interference in the religious sphere and guarantee freedom from religious laws and teachings (Feldman, 2005). People striving for secular state accept PGD because in their opinion state laws should no be influenced by religious principles (Di Marco et al., 2018; Hichy and Di Marco, 2014); then, we hypothesized that secularism should be positively correlated to the favor for PGD. With regard to the relationship between religion and secularism, studies showed that a strong identification with a religious group, as well as extrinsic and intrinsic orientations, are associated with the refusal of state secularism; while quest orientation is associated with the acceptance of the lack of religious involvement in state affairs (Hichy et al., 2015a, Hichy et al., 2015b; Hichy et al., 2014). Concerning mediating effects of state secularism, we hypothesized that the attitude toward a secular state should mediate the relationship between intrinsic orientation and attitude towards PGD. Indeed, for intrinsic religious orientation, the rejection of PGD should be due to the desire to have a state based on religious doctrines, which strive for the preservation of life. With regards to quest orientation, we hypothesized no mediating effects of secularism of state between this orientation and attitudes toward PGD; indeed, quest orientation should make people assume that medical procedure made for guarantee children's health must be conceded regardless of the secular or religious character of the state. Finally, with regards to extrinsic religious orientation, we already hypothesized that it would not correlate with attitude toward PGD; however because an indirect effect could be found even if the direct effect is not significant (Hayes, 2009; Rucker et al., 2011), is still possible that state secularism mediates the relationship between extrinsic orientation and attitude towards PGD.

## 2 Context of the study

In Italy, where the study was conducted, the majority of the population is Catholic (about 74,4%; IPSOS, 2017) and secularism is ratified by the constitution (“The State and the Catholic Church are independent and sovereign, each within its own sphere,” Article 7). As for Italian legislation about the pre-implantation genetic diagnosis of embryos, law 40/2004 (promulgated by the then right-wing government) forbids it and requires that all extracorporeal embryos produced are implanted, prohibiting their destruction or freezing, except under extremely restricted conditions. During 2005, the referendum for the partial abolition of this low was invalidated, because the voter turnout was below the 50% threshold. Despite this law, during the last years, the legal Italian context regarding PGD became more liberal, indeed, various local and higher courts approved PGD to prevent the transmission of serious hereditary diseases (see, for example, Constitutional Court 151/2009, TAR Lazio 398/2008).

## 3 Methods and material

### 3.1 Participants and procedure

Participants consist of a convenience sample of 312 Catholic Italians (174 female and 138 male) aged between 18 and 70 years (Mean = 38.50, DS = 13.63), individually recruited in various places (e.g., bus station, post office). At the end of the questionnaire, participants were asked to report their religious affiliation and their country of birth and residence; only participants who said they were Catholic and who were born and currently lived in Italy were included in the sample. All participants were informed that their responses would remain private. Ethical approval for this research was granted by the principal investigator's institution (Internal Ethic Review Board of the Department of Education Science, University of Catania). Raw data and questionnaire presented in this paper are available in Hichy et al. (2020).

### 3.2 Measures

#### 3.2.1 Religious orientations

To assess religious orientations, 14 items derived from the Religious Orientation Scale (Allport and Ross, 1967) and the Religious Life Inventory (Batson et al., 1993) were used (see also, Voci et al., 2017). The items reflect the three religious orientations: extrinsic (four items, e.g., “A primary reason for my interest in religion is that my Church is a congenial social activity”), intrinsic (six items: e.g., “I try hard to carry my religion over into all my other dealings in life”), and quest (four items: e.g., “For me, doubting is an important part of what it means to be religious”). Participants answered on a 7-point scale ranging from 1 (*absolutely false*) to 7 (*absolutely true*), with 4 meaning *neither true, nor false*. Reliability for extrinsic, intrinsic, and quest orientations was .68, .89, .73, respectively.

#### 3.2.2 Secularism of State Scale

In order to measure attitudes towards secularism, the Secularism of State Scale (Hichy et al., 2012) was used. This scale consists of eight items such as the following: “The Church should remain in its place and avoid getting involved in political affairs” and “I think it is appropriate that the Church gives its opinion on State laws” (reverse coded). For each item, participants expressed their opinion on a 7-point scale ranging from 1 (*strongly disagree*) to 7 (*strongly agree*), with 4 meaning *neither agree, nor disagree*. The reliability of the Secularism of State Scale was high (Alpha = .87).

#### 3.2.3 Attitudes towards pre-implantation genetic diagnosis

Four statements, already used in other studies (Hichy and Di Marco, 2014), were used to measure attitude towards pre-implantation genetic diagnosis (e.g., “It is right to perform pre-implantation genetic diagnosis”). Participants answered on a 7-point scale ranging from 1 (*strongly disagree*) to 7 (*strongly agree*), with 4 meaning *neither agree, nor disagree*. Alpha was .81.

## 4 Results

### 4.1 Preliminary analyses

With the aim of testing the factor structure of the scales used in this study, confirmatory factor analyses were performed (LISREL 8; Jöreskog & Sörbom, 1996–2001). To verify the acceptability of the models, we used the χ^2^: a solution fits the data well when χ^2^ is non-significant (*p* > .05). However, because the χ^2^ is affected by sample size, the two-index strategy (Hu and Bentler, 1999) was used. This strategy uses the combination of the Comparative Fit Index (CFI must be greater than or equal to .95; Bentler, 1990) and the Standardized Root Mean Square Residual (SRMR must be smaller than or equal to .08; Bentler, 1995). With regard to religious orientations results showed that three-factor structure fits the data well: χ^2^ (74) = 191.78, *p* < .001, CFI = .96, SRMR = .066; moreover, all factor loadings were significant and comprised between .45 and .88. Regarding secularism of state and attitude toward PGD, results showed that in both case one-factor structure fit the data well [for seculars of state: χ^2^ (20) = 97.31, *p* < .001, CFI = .96, SRMR = .052; moreover, all factor loadings were significant and comprised between .41 and .78; for attitude toward PGD: χ^2^ (2) = 4.71, *ns*, CFI = .99, SRMR = .019; moreover, all factor loadings were significant and comprised between .57 and .85].

The results presented in Table 1 showed that all three orientations appear to be moderate, however, the religious orientation with the highest score was the intrinsic one, followed by quest, and extrinsic [*t*_s_ (311) > 2.39, *p*_s_ < .02]. Moreover, participants were slightly favorable to a secular State and PGD. Regarding relations between variables, results showed in Table 1 indicated that extrinsic orientation positively correlates with intrinsic orientation and negatively with attitude toward a secular state; no correlation was found between extrinsic orientation and quest orientation and attitude toward PGD. Intrinsic orientation negatively correlates with quest orientation, state secularism, and attitude toward PGD. Quest orientation positively correlates with the secularism of state, while no correlation was found between quest orientation and attitude toward PGD. Finally, state secularism positively correlates with attitude toward PGD.

**Table 1:** Descriptive statistics and correlations.

		Mean	S.D.	1	2	3	4	5
1	Extrinsic	3.82	1.39	1				
2	Intrinsic	4.56	1.65	.47∗	1			
3	Quest	4.25	1.42	.02	-.12∗	1		
4	Secularism of state	4.41	1.37	-.48∗	-.63∗	.13∗	1	
5	Attitude towards pre-implantation genetic diagnosis	5.08	1.58	.05	-.32∗	.09	.26∗	1

∗*p* < .05.

### 4.2 Effect of religious orientation and state secularism on attitude towards PGD

With the aim of testing the mediating effects of secularism of State, the procedure proposed by Hayes (2009; Hayes, 2013) was used. Following this procedure, three regression analyses were conducted: 1) the mediator variable (secularism of State) was regressed on the independent variables (the three religious orientations: extrinsic, intrinsic, and quest); 2) the dependent variable (attitude towards pre-implantation genetic diagnosis) was regressed on the independent variables; 3) the dependent variable was regressed simultaneously on both the mediator and the independent variables. The indirect effect is obtained by the first and third regression, the direct effect is obtained by the third regression, and the total effects were estimated by the second regression. All regressions were carried out on 5,000 bootstrap samples randomly generated using random sampling with replacement (Hayes, 2009; Preacher and Hayes, 2008). Table 2 shows the estimates and the 95% bias-corrected confidence intervals. The indirect effect is statistically significant when the lower and upper bound of the confidence intervals do not include zero (Hayes, 2009; Preacher and Hayes, 2008).

**Table 2:** Mediating effects of secularism of State. Dependent variable: Attitude towards pre-implantation genetic diagnosis.

	Secularism of State	Attitude towards pre-implantation genetic diagnosis		Indirect effect	Bootstrapping Bias Correct 95% Confidence Interval	
	*B* (*SE*)	*B* (*SE*)	*B* (*SE*)	*B* (*SE*)	Lower	Upper
Extrinsic	-.241∗∗∗ (.047)	.282∗∗∗ (.068)	.333∗∗∗ (.070)	-.051 (.023)	-.108	-.014
Intrinsic	-.419∗∗∗ (.040)	-.408∗∗∗ (.057)	-.319∗∗∗ (.066)	-.089 (.038)	-.170	-.021
Quest	.088 (.041)	.044 (.059)	.025 (.058)	.019 (.016)	.000	.059
Secularism of state			.213∗∗ (.082)			
*R*^2^	.45	.15	.17			
*F*	84.12	18.27∗∗∗	15.71∗∗∗			
*Df*	3,308	3,308	4,307			

Note. B = unstandardized coefficient; 5,000 bootstrap samples. ∗*p* < .05; ∗∗*p* < .01; ∗∗∗*p* < .001.

Results showed that state secularism was negatively correlated with both intrinsic and extrinsic religious orientations: considering religion as the main reason in life, as well as using religion for utilitarian reasons, are associated with the refusal of state free from religious values.

Regarding attitude towards PGD, results showed that the correlation with extrinsic orientation was positive while that with intrinsic orientation was negative: living the life following religious principles is associated with the refusal of the use of PGD while using religion only to satisfy other needs is associated with the acceptance of the use of PGD. It should be noted that, using bivariate correlations, no correlation was found between extrinsic orientation and attitude toward PGD; while, controlling for other religious orientations the relation between extrinsic orientation and attitude towards PGD is positive. With the aim to understand which orientation between intrinsic and quest influence the relationship between extrinsic orientation and attitude towards PGD, partial correlations were carried out. The correlation between extrinsic orientation and attitude toward PGD was positive and significant when controlling for intrinsic orientation (*r*_ab.c_ = .24, *p* < .001) and not significant when controlling for quest orientation (*r*_ab.c_ = .05, *ns*). These results may suggest that the positive correlation found between extrinsic orientation and attitude toward PGD was due to the effect of intrinsic orientation, that is, it is possible that controlling for real endorsement in religion, people that use religion for other aims are favorable to PGD.

With regard to the effect of attitude towards a secular State, results showed that it is positively correlated with attitudes towards PGD: the more favorable people are to secular state the more favorable they are toward PGD. Moreover, results showed that secularism of state partially mediated effects of both intrinsic and extrinsic religious orientation, indeed, the confidence interval did not include zero, and the direct effect of both religious orientations on attitude toward PGD was still significant controlling for attitude towards State secularism. These results indicate that real religiosity is associated with the rejection of PGD because state laws should follow religious principles; while the use of religion to reach other aims is associated with the acceptance of PGD because state laws should mirror religious principles.

## 5 Discussion

In this study, we tested the effects of attitude towards secular State and religious orientations on attitudes towards the pre-implantation genetic diagnosis of embryos. Concerning religious orientations, our results suggest that strong intrinsic religious orientation is associated with the observance of religious norms, which, in the case of the Catholic religion, condemned PGD, because during diagnosis some embryos might be discarded and, then, destructed, committing a sin for the Catholic religion. This result confirms those of other studies suggesting that people that strongly endorse religion reject practices that violate the religious teachings and values, because considered as “sins” (Bosetti et al., 2011; Mak and Tsang, 2008).

Regarding extrinsic orientation our results are counterintuitive, indeed they indicate that extrinsic religious orientation is associated with the acceptance of PGD when controlling for intrinsic orientation. This could mean that once eliminating real endorsement in religion, utilitarian aspects of religion are associated with favor towards PGD. Indeed, because extrinsic religious orientation involves the use of religion to reach other aims, the PGD may be seen, even if it is against the religious prescriptions, as a mean to reach the aim to have healthy children. This result confirms the component of extrinsic religious orientation related to compartmentalization, namely the ability to separate the religion from the rest of life (Batson and Schoenrade, 1991).

Finally, regarding quest orientation results showed no relationship with attitudes towards PGD. One possible explanation is that the knowledge that religion does not have all answers, do not let religious beliefs influence people's attitude towards scientific technologies. However, this point deserves further investigation.

Whit regard to attitude towards a secular State, results showed that it is positively associated with attitudes towards PGD. Persons who are favorable to secular state believe that state laws should be free from religion and religious prescription influence, and therefore are favorable towards PGD, even if it is not allowed by religion. With regard to the mediating effects of secularism on the relationship between religious orientations and attitude towards PGD, results showed that secularism mediated the effects of intrinsic and extrinsic religious orientations. These results indicate that a sincere belief in religion is associated with the rejection of PGD because state legislation should follow religious principles. Concerning extrinsic orientation, results suggested that the use of religion to satisfy self-interest is associated with the acceptance of PGD because a secular state is rejected. This almost paradoxical results can be explained through the instrumental role that, for extrinsic orientation, the religion has. For extrinsic orientation religion only serves to satisfy self-interest and other personal needs, and religious doctrines are used and shaped to adapt to needs satisfaction. In this sense, the extrinsic orientation is associated with the acceptance of PGD because people want to maintain a state that reflects religious principles and ensures high status and privileges for the Catholic group. It is like the acceptance of a couternormative procedure might help to maintain the status quo. This kind of behavior looks like some kind of defensive help (Nadler, 2002), whereby PGD is accepted with the aim to mitigate the threat to the Catholic group's status derived form a secular state; indeed, defensive help is more likely to be used when the group status is threatened. Same results of extrinsic orientation were found in relation with gay and lesbian civil rights (Hichy et al., 2015a, Hichy et al., 2015b), indeed also in the case of this other issues going against religious principles, the extrinsic orientation makes people willing to go against religious principles - accepting same-sex marriage and adoption by same-sex couples - while maintaining the status and privileges that are guaranteed to them by a non-secular state.

## 6 Conclusion

The results of this study identify important aspects of attitudes related to PGD. With regard to religion, we find that a poor indicator of religiosity (e.g., religious attendance or religious practice) is not able to completely explain the relationship between religiosity and attitude towards PGD and confirmed the importance of distinguishing between dimensions of religiosity (Batson et al., 1993). Indeed, we find that the intrinsic dimension plays an important role in the rejection of PGD while the extrinsic dimension contributes to the acceptance of PGD. Moreover, our results confirm the utilitarian aspect of extrinsic religious orientation and its emphasis on materialistic goals such as success and status (Spilka, 1977): the lack of sincere involvement in religion involve ignoring its dictates and accepting PGD.

As for attitude towards a secular state, we find that the desire to have a state less influenced by religion is an important variable in investigating attitude towards PGD, indeed the acceptance of PGD is related to the desire of absence of religious involvement in state affairs. Then, as in the case of other important issues, such as abortion or gay and lesbian civil rights (Hichy et al., 2015a, Hichy et al., 2015b; Tamney et al., 1992), this study confirms the important role played by secularism and religion in decision making. Moreover, regardless the specific attitude investigated in this study, the link between religion and secularism may have more general implication on the society; indeed, the rejection of secularism supported by intrinsic and extrinsic religious orientations can undermine the equality and the rights that a state should guarantee to all citizens.

The results of this study may be useful for policymakers, but also for the general public, to make clear the role played by religion in influencing public attitude towards PGD. Moreover, these results may be useful to promote public campaigns aimed to make people aware that in order to guarantee equal rights of all citizens, as well as, to prevent the diffusion of serious illness in population and promote scientific health progress, state legislation should not be influenced by religious beliefs. Indeed, in a secular state, such as Italy, legislation should not be influenced by religion.

## 7 Limitations and further investigations

A major limitation of this study is its correlational nature, indeed it is not able to establish a clear causal relationship between the variables. Further studies should use and experimental or at least a longitudinal design. Moreover, this study did not take into account the effects of knowledge about PGD that may influence the attitude toward it (Meister et al., 2005). Another limitation of this study is the sample: indeed, recruiting only willing respondents may introduce a bias. Moreover, this study was conducted in Italy, where secularism of the State, even if it is ratified by the constitution, is affected by the vicinity to Pope and Vatican City (Di Marco, 2009). Indeed, in the case of the 2005 referendum, evaluating the partial abolition of law 40/2004, the then Pope Benedict XVI discouraged Italians from going to vote, invalidating the results because the voter turnout was below the 50% threshold (Fisher and Povoledo, 2019). Although Italy, due to the characteristics mentioned above, is a context of choice for the analysis of the constructs investigated in this study, further research should explore the effects of state secularism in other countries (e.g., Spain, where Catholicism is the most widespread religion, but that could be less affected by the nearness of Vatican City) and/or with other religious groups. In particular, the interplay between extrinsic orientation and secularism needs further study, to confirm or disconfirm those obtained in this study. Finally, concerning secularism, in this study we considered it as a general separation between church and state, however, as highlighted in some studies secularism may assume different forms (Roebroeck and Guimond, 2016) and could be interesting understand which form of secularism affects attitude involving procedure, such as PGD, or other issues, such as same-sex marriage, that religion refuses.

## Declarations

### Author contribution statement

Z. Hichy: Conceived and designed the experiments; Performed the experiments; Analyzed and interpreted the data; Contributed reagents, materials, analysis tools or data; Wrote the paper.

F. Sciacca, G. Di Marco, C. De Pasquale: Analyzed and interpreted the data; Contributed reagents, materials, analysis tools or data; Wrote the paper.

### Funding statement

This research did not receive any specific grant from funding agencies in the public, commercial, or not-for-profit sectors.

### Competing interest statement

The authors declare no conflict of interest.

### Additional information

No additional information is available for this paper.
